# Differentiating Use of Facial Expression between Individuals with and without Traumatic Brain Injury Using Affectiva Software: A Pilot Study

**DOI:** 10.3390/ijerph20021169

**Published:** 2023-01-09

**Authors:** Kelly Yiew, Leanne Togher, Emma Power, Melissa Brunner, Rachael Rietdijk

**Affiliations:** 1 Faculty of Medicine and Health, University of Sydney, Sydney, NSW 2006, Australia; 2 Graduate School of Health, University of Technology Sydney, Sydney, NSW 2007, Australia

**Keywords:** traumatic brain injury, software, facial expression, assessment tool, social communication

## Abstract

This study investigated the feasibility of using an automated facial coding engine, Affectiva (integrated in iMotions, version 8.2), for evaluating facial expression after traumatic brain injury (TBI). An observational cross-sectional study was conducted based on facial expression data from videos of participants with TBI and control participants. The aims were to compare TBI and control groups, and identify confounding factors affecting the data analysis. Video samples of two narrative tasks (personal event and story retell) from ten participants with severe TBI and ten control participants without TBI were analyzed using Affectiva. Automated data on participants’ engagement, smile and brow furrow were compared statistically between and within groups. Qualitative notes for each sample were also recorded. Affectiva detected a higher percentage of time of engagement for TBI participants than for control participants on both tasks. There was also a higher percentage of time of smiling for TBI participants in one task. Within groups, there were no significant differences between the two narrative tasks. Affectiva provides standardized data about facial expression and may be sensitive to detecting change in the use of facial expression after TBI. This study also identified factors to avoid during videorecording to ensure high quality samples for future research.

## 1. Introduction

Social communication deficits are common after people sustain a traumatic brain injury (TBI) [1]. These communication difficulties are at the level of discourse often beyond words and sentences so that people with TBI may struggle to communicate well in social and conversational contexts but show no marked deficits in speech or language [2].

A successful conversation is dependent on the content of discourse and the non-verbal aspects of communication, including the ability to interpret and use facial expression. Ruusuvuori and Peräkylä [3] noted that facial expression is a resource both for the person and their conversation partners, and it serves in the construction of meaning and formation of relationships between them. People with TBI have difficulties identifying people’s emotions based on situational and contextual cues [4,5]. Furthermore, difficulty using facial expressions after TBI can also impair their ability to react in socially appropriate ways to different communication situations [6]. Deficits in the interpretation and use of facial expression could lead to communication breakdown, and consequently, difficulty with social integration [7,8]. As a result, people with TBI may be perceived as less socially competent [9,10], and interactions with them regarded as less enjoyable and rewarding [9]. This breakdown in social communication can be a barrier in the formation and maintenance of relationships [11,12,13,14] and can be associated with decline in social networks, strain on families, and disruption to social lives [14,15,16].

While the impact of difficulty in interpreting facial expressions on social communication after brain injury is well recognized, the ability to use facial expression successfully is less well understood. For some individuals with TBI, facial expression during social communication is impaired, including a lack of expression [17], or using facial expression inappropriately due to disinhibition [6,18]. One study found that people with TBI had difficulty adapting their facial expressions to show different emotions while reading neutral sentences in a structured assessment task, as compared to people without a TBI [19]. However, there is limited research in this field, and it is important to investigate the use of facial expression to better understand the profile of impairments after TBI that could be targeted during rehabilitation to support more successful and enjoyable conversations. Given that the use of facial expression during communication will be affected by context, it is also relevant to explore whether different communication tasks elicit greater display of expression, whether negative or positive. For example, speakers retelling a significant injury or illness may use facial expression to underscore the seriousness of the event. A retelling of an impersonal narrative, such as a fairytale, may have less emotional weight, but speakers might still use facial expression for dramatic effect to engage the listener. Therefore, the use of facial expression in different communication contexts is an area worthy of further investigation, which could help develop future research and clinical tools for people with TBI.

There are standardized assessment measures to evaluate recognition of facial expression, such as The Awareness of Social Inference Test (TASIT) [20]. However, there are no standardized measures to evaluate the use of facial expression after brain injury. Existing tools for evaluating non-verbal communication of people with TBI, including facial expression, are based on the subjective judgment of the speech pathologist, and involve global ratings, such as whether a behavior is appropriate or inappropriate [21,22]. The development of technology has allowed automated recognition of facial expressions during video samples of conversation, which could provide more specific information about how people with TBI use facial expressions. Affectiva is a potential, and yet untapped, automated software analysis resource that may be beneficial in evaluating the use of facial expression by people with TBI. Affectiva’s AFFDEX [23] is a software program integrated into the platform called iMotions (Version 8.2). iMotions combines and synchronizes different types of biosensors such as eye tracking, facial expression analysis, electrodermal activity, from various independent vendors into a single platform [24]. Affectiva is the automated facial coding engine used by iMotions for facial expression analysis. The software has a coding system which analyzes facial expressions based on processing video samples using automated computer algorithms [25]. It measures ‘7 core emotions—joy, anger, fear, disgust, contempt, sadness, and surprise’ through 20 facial action units [25]. Affectiva has been measured against results produced by facial electromyography, and demonstrated validity, with high correlations, in identifying facial expressions and related emotions [26]. Affectiva has been used in previous clinical studies examining facial expression of individuals with autism and individuals with dementia in Parkinson’s Disease. In autism research, Affectiva was integrated into an emotion recognition game to provide automatic recognition and evaluation of users’ emotions from facial expression [27]. In a study focusing on dementia in individuals with Parkinson’s Disease, Affectiva was used to quantify the participants’ difficulty in facial expression imitation tasks [28]. These studies indicate that Affectiva may be useful to evaluate facial expression in clinical populations, but this software has not previously been applied to research in TBI. Furthermore, the feasibility of using Affectiva to evaluate use of facial expression during communication tasks has not been investigated.

### Aims

This study investigated the use of Affectiva as a potential objective measure of facial expression during communication in speakers with TBI to answer the following research questions:

1. Were there any differences in the use of facial expression between TBI participants and controls during narrative tasks using Affectiva?
2. Were there any differences in the use of facial expression between a personal event narrative task and a story narrative retell task?
3. Were there any confounding factors in video samples that may influence the validity or feasibility of the Affectiva analysis?

## 2. Materials and Methods

### 2.1. Study Design

An observational, cross-sectional study was conducted using previously collected video samples from an existing database namely TalkBank [29]. The original data was obtained within ethical protocols. TalkBank had received informed consent release from the individual participants when the data was collected. The participants understood that the data collected would be used by researchers and educators. As Affectiva has not previously been used for evaluating the use of facial expression during communication tasks, this was considered to be a pilot feasibility study.

### 2.2. Participants

#### 2.2.1. Selection of Participants

Participants for this study were drawn from TalkBank, specifically the TBI Bank [30] and Aphasia Bank corpora [31].TBI Bank is a databank that includes people with TBI whereas Aphasia Bank is a databank that includes controls without a brain injury and people with aphasia. The controls without a brain injury drawn from Aphasia Bank were the control group for this study. Both databanks comprise videotaped samples of participants completing standardized discourse tasks based on a structured protocol, including a personal event narrative task and a story narrative retell. Demographic data for participants who have contributed video samples is also available within these established databanks.

The inclusion criteria for selection of participant video samples were:

(a) Participants were aged between 18–65 years;
(b) Video samples were available for both a personal event narrative task and a story narrative retell. For participants with TBI, it was required that these video samples be available from their 6 month post-injury assessment.
(c) Participants had a severe TBI, with injury severity determined by length of post traumatic amnesia (PTA), whereby a person with severe injury has PTA > 24 h [32,33].
(d) Narrative tasks were spoken in English

The exclusion criterion for selection of video samples was:

(a) Participant videos that were unable to be processed sufficiently for analysis (less than 70% of the video sample processed) by Affectiva. For example, where the participant was not front-facing the camera, or poor lighting conditions.

#### 2.2.2. Matching of TBI and Control Participants

**Figure 1:**
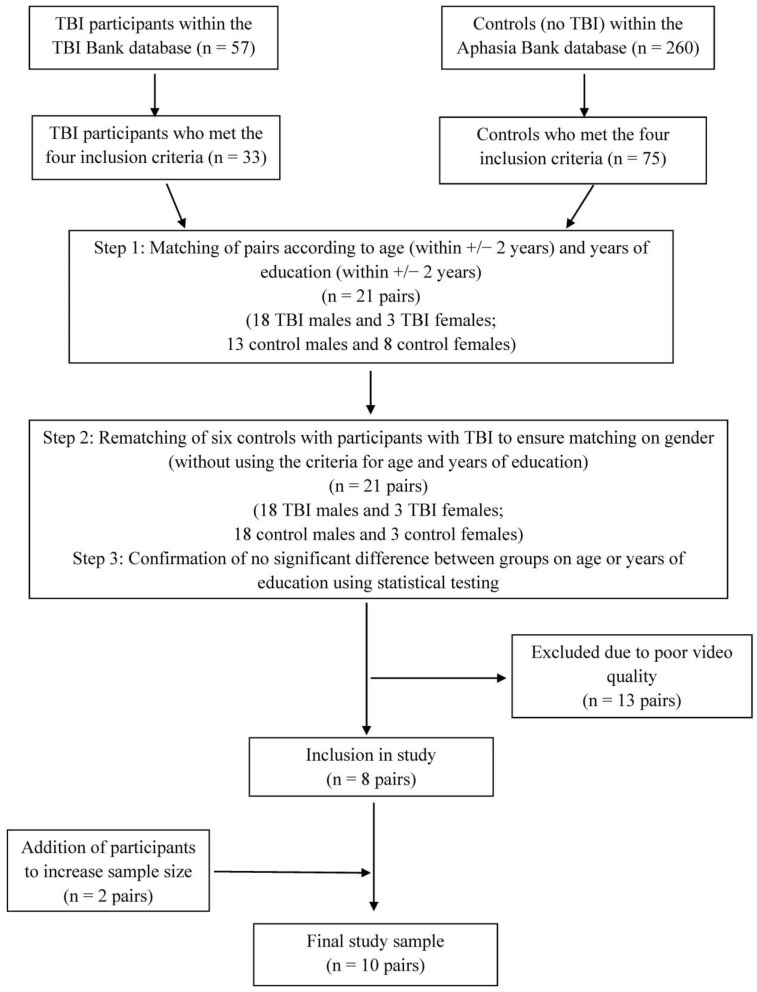
Flow diagram for study participants.

A sample of 10 participants who had a TBI and 10 participants who did not have a TBI (control participants) was included in this study, with participants matched in pairs based on demographic characteristics. Figure 1 is a flow diagram showing the criteria and process for matching participants. Participants were first selected according to the four inclusion criteria. Participants with TBI were then matched with controls according to gender, age, and years of education following a two-step process as shown in Figure 1. Matched pairs in which either participant had samples which did not meet the criterion for video quality were excluded. The matching process created a cohort of eight matched pairs. To increase our sample size to ten matched pairs, we repeated the matching process according to our inclusion, exclusion, and matching criteria and identified a further two matched pairs by re-matching individuals with adequate video quality from the originally excluded pairs.

#### 2.2.3. Participants with TBI

There were nine males and one female, with ages ranging from 24 to 54 years (mean = 37.8, SD = 10.63), and years of education ranging from 10 to 18 (mean = 14.5, SD = 2.88). All participants resided in Australia at the time of data collection. All participants had severe TBI, with PTA ranging from 14–103 days. Table 1 below presents further information on the participants with TBI.

#### 2.2.4. Controls

The control group comprised of nine males and one female, with ages ranging from 23 to 57.5 years (mean = 38.3, SD = 11.42), and years of education ranging from 12 to 18 years (mean = 15.3, SD = 2.11). All the participants in the control group resided in the United States at the time of data collection and had no history of stroke, head injury, neurological condition, or communication disorder. Table 2 below presents further information on the controls.

#### 2.2.5. Comparison between Groups

Comparison between groups demonstrated the TBI and control groups were well-matched. The gender distributions in both TBI and control groups were the same (nine males and one female). There were no significant differences statistically between the two groups for age (*t* = 0.09, *p* = 0.93) or years of education (*t* = 0.71, *p* = 0.49).

### 2.3. Video Samples

#### 2.3.1. Narrative Tasks

This study focused on two narrative tasks, which were personal event narrative and story narrative retell. Narrative story telling is a well-researched discourse type [34] commonly used in daily conversations [35]. Stark [36] found that narrative discourse elicited the densest language as compared to picture description and procedural discourse (with procedural discourse eliciting the shortest mean length of utterance). It was of interest to compare two different narrative discourse tasks, to determine whether speakers with or without a TBI adapt their use of facial expression for different contexts. For example, it is possible that the personal event narrative, about a significant illness or injury, may elicit more use of facial expression than a story narrative retell task.

Elicitation of the narrative discourse tasks was undertaken by qualified speech pathologists who used standardized protocol instructions and scripts [30,31].

(a) Personal event narrative

Participants with TBI and controls were asked to talk about their brain injury and an episode of personal illness, respectively. Step-by-step instructions were given to the examiner to follow. The elicitation prompts are as follows:

i. For participants with TBI, “Tell me what you remember about when you had your head injury”.
ii. For controls, “I wonder if you could tell me what you remember about any illness or injury you’ve had”.

When the participants had difficulties providing responses, the examiner would proceed to use troubleshooting questions.

(b) Story narrative retell

Story narrative was elicited using the ‘Cinderella’ story. The person was asked to tell the story of Cinderella, following the viewing of a wordless picture book. Similar to the personal event narrative, the examiner provided elicitation prompts according to the protocol instructions. If the participant had any difficulty, the examiner would prompt further for more information.

For the TBI participants, video samples ranged in length from 00:16 to 02:08 min for the personal event narrative task, and 00:56 to 03:33 for the story narrative retell task. For the control participants, video samples ranged in length from 00:22 to 03:01 for the personal event narrative task, and 01:03 to 05:31 for the story narrative retell task. There was no significant difference (*p* = 0.052, *U* = 24.00) between the TBI and control groups on the personal event narrative. The TBI group tended to speak for a shorter duration (median = 00:34) as compared to the control group (median = 01:20). For the story narrative retell task, there was a significant difference (*t* = 2.64, *p* = 0.02) between the duration of video samples of TBI (mean = 02:22, SD = 00:54) and control (mean= 03:44, SD = 01:21) participants.

#### 2.3.2. Affectiva Analysis

**Figure 2:**
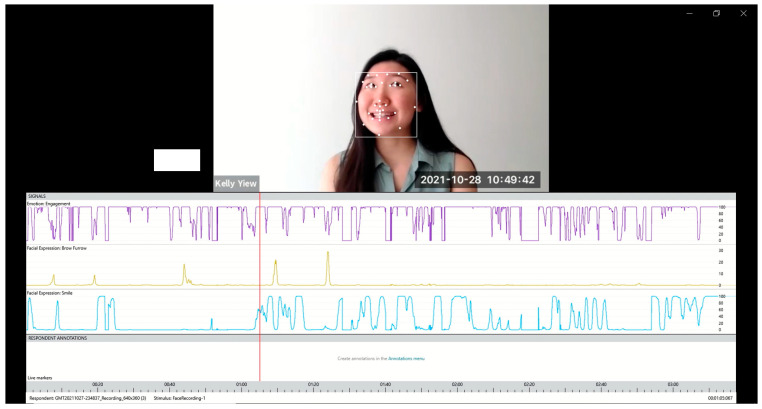
Screenshot demonstrating Affectiva functions.

The last author completed two, one-hour iMotions onboarding sessions to learn about the functionality of the platform and the analysis, and then provided orientation training to the first author. Videos extracted from TBI Bank and Aphasia Bank were edited by the first author to create video samples for analysis. Video samples commenced from when the participant started speaking and finished when the participant stopped speaking. Any verbal prompts from the examiner were cropped out of the sample. The first author then imported the video samples into the iMotions platform and analyzed them using the automated facial coding engine Affectiva. According to iMotions’ facial expression analysis guidebook [23], automatic facial coding consists of three elemental steps: face detection, feature detection, and feature classification. An example demonstrating the three steps of automatic facial coding in Affectiva is shown in Figure 2. In face detection, the face is detected and framed in a box. The next step is feature detection, where facial landmarks (e.g., eyes, brows, and mouth corners) are detected (as marked by dot points in Figure 2) and adjusted according to scale. Lastly, in feature classification, information on the key features is obtained and inputted into the classification algorithms. The program then translates the features into facial expression metrics, represented in the three traces, with engagement, brow furrow, and smile being analyzed in Figure 2.

#### 2.3.3. Facial Measures

This study focused on three facial measures, which were engagement, smile, and brow furrow. When using Affectiva, engagement is described as a measure of facial muscle activation that displays the individual’s expressiveness [37]. ‘Engagement’ was a relevant measure as it allowed us to examine the overall facial animation of the participants, which we anticipated would provide an overall measure of whether speakers were displaying flattened or heightened affect during the discourse sample. ‘Smile’ and ‘brow furrow’ were selected as facial expression measures to include measures associated with positive and negative emotions, respectively. These two expressions also have a high accuracy of detection [37]. A low threshold of 2/100 (representing a 2% probability that the expression was present) was set to ensure that all data was presented in order to assess the data optimally. Time percentages for engagement, smile, and brow furrow across the participants’ interactions were generated by Affectiva. The use of time percentages in the analysis of the measures controlled for the differences in sample duration between the groups.

#### 2.3.4. Qualitative Observation

Qualitative notes for the video samples were recorded by the first author using an observation guide. Table 3 shows an example of how the qualitative observations were recorded. A frequency rating scale of 1 to 5 was used to rate the video samples to provide an overall, global clinical impression of the frequency of facial expression during a video sample. A rating of 1 represented the participant never showed any signs of the specific facial measure, 2 represented rarely, 3 represented sometimes, 4 represented often, and 5 represented always. This simple scale provided a clinician-driven gauge of the frequency of the participants using their facial expressions according to the facial measures, to categorise whether they were overly expressive or having a flat affect. This process also guided the analysis of our results as we reviewed Affectiva outlier data by comparing it against our qualitative observations. Clinical impressions included any other observations such as pragmatics and discourse. An overall impression of whether the participant’s interaction was socially appropriate was recorded. To reduce bias on clinical impressions, the qualitative observation process was conducted before statistical analysis of the Affectiva data.

To develop consensus between study authors in the use of the observation guide, 13 video samples were selected at random (33%) from both TBI and control groups to be clinically analyzed between all authors. The authors included a student speech pathologist and four speech pathologists experienced in the field of TBI rehabilitation. The authors first rated the video samples independently. Consensus discussions were then held to establish agreement of the ratings between the authors, with qualitative notes on the clinical contexts and clinical impressions also discussed. Once consensus regarding the procedure was established, the first author then independently rated the remaining samples.

### 2.4. Data Analysis

Data analysis was supported using SPSS statistical software (IBM SPSS, Version 26). Firstly, the duration of the video samples for the personal event narrative and story narrative retell tasks were compared to identify any differences between the two tasks. Independent samples *t*-tests were used for normally distributed data and independent-samples Mann–Whitney U tests were used for non-normally distributed data.

For research question 1, independent-samples Mann–Whitney U tests were conducted to compare data between TBI and control participants for each narrative type. For research question 2, related-samples Wilcoxon signed rank tests were conducted to determine whether there were significant differences within the two different narrative types for the TBI participants, and for the control participants. The Bonferroni correction was considered but due to the need guard against type 2 errors in early pilot research, the correction was not applied [38,39]. For research question 3, samples where outliers were identified in the Affectiva data were examined as case studies to explore any confounding factors.

## 3. Results

### 3.1. Affectiva Data

Data for individual participants are reported in Table 4 and Table 5. To address research question 1, statistical comparisons between participants with TBI and controls for the personal event narrative and story narrative retell are presented in Table 6 and Table 7, respectively.

When examining the results for the personal event narrative, there was a significant difference (*p* = 0.011, *U* = 82.50) in engagement between TBI participants and controls but not for smile or frown. Higher engagement was detected for participants with TBI (median = 47.53) compared with control participants (median = 1.96).

For the story narrative retell task, there was a significant difference (*p* ≤ 0.001, *U* = 93.00) was observed in engagement between TBI and control participants, with higher engagement for TBI participants (median = 54.73) than for control participants (median = 5.98). There was also a significant difference in smile (*p* = 0.0015, *U* = 82.00) between the groups, with a higher median time percentage detected for TBI participants (18.23) compared to control participants (0.32). No significant difference between groups was observed in brow furrow.

To address research question 2, statistical comparisons were conducted to compare data within TBI and control participants for the personal event narrative and story narrative retell. Table 8 and Table 9 display the median of differences between the facial measures in the TBI and control participants, respectively. There were no significant statistical differences observed between the facial measures within the TBI group and control group on the two different discourse genres.

### 3.2. Qualitative Observations

Qualitative observation data is presented in Appendix A. For engagement and smile, the ratings ranged between 1 and 4 for the participants with TBI. The control participants had the same range of scores for engagement and smile. For brow furrow, ratings ranged from 1 to 3 for the participants with TBI, and the control participants had the same range of scores. Both TBI and control groups were judged as presenting with socially appropriate use of facial expression, with only one participant with TBI presenting with less appropriate use of facial expression during the narrative tasks. This participant was observed to have a fixed facial expression throughout the narrative tasks, and a raised brow when speaking.

### 3.3. Case Studies

To address research question 3, samples with extreme data points from the Affectiva analysis (i.e., zero values for all measures, or with data identified as outliers using a box and whisker plot) or unusual values (i.e., participants with highly contrasting data for the two tasks) were identified. Seven out of the 40 samples had extreme data points, and one participant with TBI had highly contrasting data between the tasks. These identified samples were then further explored by comparing data from Affectiva and qualitative observations. We also consulted specialists from iMotions to review the data generated from Affectiva to examine plausible factors resulting in the outliers. To illustrate these findings and our interpretations, case studies are presented below where zero values, outlier data, and divergent data were observed for individual participants.

#### 3.3.1. Case Study 1—Zero Values

The data as generated by Affectiva showed that TBI4 and C8 during the personal event narrative task recorded 0 for all measures. Review of these samples showed that the participants were not directly front-facing the camera. It seemed that they were facing the interviewer during the task instead. This could have impacted Affectiva’s ability to accurately analyze the video samples. Given these participants did have facial expressions detected in the story narrative retell, the video samples for these two participants were compared. We found that in the story narrative retell video sample of C8, their whole face could be seen. However, only half of their face was captured in the personal event narrative task. This could potentially contribute to the outlier data during the personal event narrative task as Affectiva might not be able to accurately analyze the participant’s face when they are side-facing the camera. On review of the two samples for TBI4, there were no observable differences in the participant’s positioning. However, a possible confounding factor present in both samples was the participant’s glasses. It appeared that in the story narrative retell task, TBI4’s eyes were blocked by the frame of their glasses, whereas their eyes were not blocked by glasses in the personal event narrative task

#### 3.3.2. Case Study 2—Outlier Data

Results identified as outliers are represented by dots on the box and whisker plots (refer to Appendix B). Samples with data identified as outliers were C2, C3, C10 from the personal event narrative task, and TBI2 and C2 from the story narrative retell task. These video samples were reviewed again using iMotions to identify any factors impacting detection of participants’ facial landmarks. There was evidence that the use of accessories interfered with Affectiva’s facial detection. In C3’s video sample during the personal event narrative task, Affectiva was detecting the participant’s hat in some instances. The participant was also slightly side-facing the camera, thus occasionally causing the facial detection to get lost momentarily. Another possible factor that could have contributed to the outliers was the distance between the camera and the participants. Upon review of the video samples, we concluded that some outliers could be explained by the facial features of the participants. For example, there was one participant with a much higher brow furrow time percentage than others, as he had an observable furrow on his forehead as part of his usual expression.

#### 3.3.3. Case Study 3—Divergent Data between Narrative Tasks

TBI10 had divergent data between the personal event narrative task and story narrative retell task for engagement, with the second lowest engagement data in personal event narrative (4.03) but the second highest engagement data in the story narrative retell (85.53). Upon reviewing the video samples of TBI10 across both narrative tasks, a potential reason that could have impacted our data is the change in use of glasses. In the personal event narrative task, TBI10 did not wear glasses, but in the story narrative retell task TBI10 wore glasses. Similar to case study 1, this suggests that the use of glasses could have influenced Affectiva analysis, thus generating a difference in engagement value.

## 4. Discussion

Use of facial expression is an important aspect of everyday communication that may be affected following severe TBI, leading to impaired social communication outcomes [8]. Facial expression has not previously been measured in a quantified and reliable manner, with a reliance on subjective impressions or ratings on pragmatic communication protocols such as the Profile of Pragmatic Impairment in Communication [22]. This is the first research conducted to investigate the use of Affectiva as a potential objective measure of facial expression in speakers with TBI compared to those without TBI. For research question 1, the Affectiva data showed some differences between the use of facial expression between the TBI and control groups. There were significant differences in engagement scores on Affectiva between TBI and control participants across both narrative tasks. This preliminary data suggests that the global measure of engagement (which is a composite measure across multiple expressions) may have more potential to demonstrate differences between TBI and control group as compared to specific action units of smile and brow furrow. A significant difference between groups in the smile time percentage was detected during the story narrative retell, but not in the personal event narrative. It is possible that the story narratives had a more consistent emotional tone across participants, compared to the personal narratives, which may have been either a positive or negative topic. The higher degree of structure in the story narrative task may make this task more suitable for future group studies investigating the use of facial expression during communication. There were no significant differences in the use of brow furrow detected between groups in this pilot study.

This study also investigated whether any differences were detected in the use of facial expression between the two different narrative tasks, to understand if speakers differentiated their use of facial expression between these two genres. From our investigation, there were no differences in the use of facial expression between a personal event narrative task and a story narrative retell task. This finding provides preliminary evidence that speakers with and without a TBI use similar frequency and types of facial expression when completing different narrative tasks, regardless of the topic. However, this finding should be interpreted with caution due to the small sample size in this pilot study. Further research involving comparison of topics across a larger number of participants would be needed to validate this result.

A pattern observed across both narrative tasks was that TBI participants had higher engagement, smile and brow furrow than the control participants, as evaluated by the Affectiva analysis. This result was surprising as it contrasted with previous research indicating that individuals with TBI had difficulties using facial expression to express themselves [8,19]. A possible reason for this discrepancy is that participants with TBI could have increased their use of facial expression to compensate for other factors that could be impacting their communication, for example, verbal output and memory abilities. Another factor could be the duration of the video samples. TBI participants had a shorter video sample than control participants across both narrative tasks. Momentary peaks in the use of facial expression in shorter samples would have a larger impact on the results, compared to a longer video sample. Future research in the use of facial expression during communication could ensure consistency of speaking duration across samples to address this issue.

Given the unexpected findings, it was important to review the samples to identify any confounding factors which influenced the data. This study used video samples from a databank, rather than samples collected for the specific purpose of evaluating facial expression. This provided an opportunity to observe factors in the recordings which influenced the data, so that these factors can be avoided when collecting samples for future research. From our qualitative observations, one confounding factor was that some participants were not directly front-facing the camera. As the whole face could not be detected, this may have affected Affectiva’s sensitivity when analyzing facial movements. This observation is consistent with the iMotions guidebook [23], in which it is stated that the camera should be placed approximately at the participant’s eye level and facing the participant directly. Another confounding factor which appeared to influence the data was the participant’s use of accessories such as glasses and hats. These could interfere with Affectiva’s detection of the participant’s face. Again, this is consistent with the iMotions guidebook [23], which notes that glasses covering the eyebrows and hats could occlude facial landmarks, which may lead to partial results. Beyond these expected observations, some instances of outlier data appeared to relate to Affectiva accurately detecting features of an individual’s facial structure. We observed that there were participants with a more furrowed brow even at rest, which was detected as a higher brow furrow time percentage. These individual differences in facial structures appeared to impact the results generated by Affectiva. Future research may need to include a baseline task to control for individual differences. A final consideration is the impact of the facial movements used for speaking on the Affectiva analysis. It did not appear that the speech movements were detected as expressions based on review of the video samples. However, speaking has been previously identified as an issue that can influence Affectiva analysis [23].

Aside from the factors affecting quality of the video samples, it should be noted that the automated facial coding system in Affectiva analyzes the facial expression without consideration of the context of the discourse. Affectiva is unable to identify the appropriateness of use of facial expression, such as a mismatch of facial expression to the context and content of what was said. As such, our qualitative observations took into account the context and content of what participants said before making judgments on whether their use of facial expression was socially appropriate overall. For example, a participant could score a low level of engagement, smile, and brow furrow from the Affectiva data. However, the participant’s use of facial expression could still be socially appropriate given that the context being talked about did not involve any strong emotions, thus their facial expression appropriately matched the discourse. Although it has been suggested that people with TBI have difficulty with use of facial expression [6,17,18], only one of the ten participants with TBI in this study was judged as using facial expression inappropriately on qualitative observation of the narrative samples. This suggests that further research is needed to understand the prevalence of difficulties with using facial expression after TBI.

### 4.1. Limitations

This exploratory investigation used a small sample size of 10 participants in each group, with the majority of participants being male. Future research with a larger sample size could provide greater insights and strengthen results. It would also be valuable for future research to aim for a gender balance in the participant sample, given that gender may influence use of facial expression during communication. Additionally, secondary data were used in this study. Therefore, the video samples were not optimally set up for Affectiva analysis. Although inclusion criteria were used to ensure that the selected samples could be successfully processed, there were factors identified in the samples which may have influenced the analysis. For example, some participants were side-facing the camera, and wore glasses and hats which interfered with the facial detection software that could have generated inaccurate results. Therefore, due to the small sample size and the factors affecting the video quality, a definite conclusion cannot be drawn. Lastly, even though the participants were well-matched on their demographic characteristics, the participants’ cultural backgrounds were different, with the TBI participants from Australia and the control participants were all monolingual and from the United States. Both groups completed the narrative tasks in English, but the differences in culture could have affected the data nonetheless.

### 4.2. Clinical Implications and Directions for Future Research

Although differences in the facial expression data were detected between groups, further research will be needed to understand the use of facial expression in communication situations after brain injury, and clinical implications for assessment and treatment. A clear recommendation for future research investigating facial expression is to standardize the recording set-up to optimize video samples for Affectiva analysis. It is recommended that participants are all fully front-facing the camera and not wearing any accessories that could interfere with Affectiva facial detection and analysis. It has been found that telehealth as a service delivery option has sizeable potential benefits for individuals with TBI [40], and so it may be feasible to engage participants in data collection for future studies via telehealth, following a protocol to ensure that participants are in an optimal recording environment, for example, front-facing positions, with appropriate lighting and distance to the camera. With these practices in place, Affectiva could potentially serve as an objective clinical and research tool for assessments and therapy interventions focusing on social communication. Larger studies with greater numbers of participants using standardized elicitation measures and formatting as outlined here would enable further understanding of the degree and nature of potential facial expression deficits in TBI, as currently this is not known.

With the advancement of technology, the use of automated software in other areas of speech pathology has become increasingly prevalent. For instance, there is now automated software for speech and voice analysis such as Praat [41] and Visi-Pitch [42], where traditionally analysis was dependent on the clinician’s subjective judgment during assessments and treatments. Similarly, we envision Affectiva could be used as an objective guide to support clinicians’ decision-making process in planning therapy intervention and improving outcomes. Affectiva could also be a source of data for clinicians and researchers in relation to social cognition, specifically to evaluate a person’s ability to use facial expression appropriately to interact and to respond to other people. TalkBank, which includes different corpora such as TBI Bank, Aphasia Bank, Dementia Bank, and RHD Bank, currently has automated software programs such as spoken dictation systems and automated analysis tools for language and speech available [43]. Affectiva could similarly be applied to the analysis of TalkBank data to provide a fine understanding in the use of facial expression of people with different communication disorders. It is noteworthy that the speech movements during the narrative tasks did not appear to be a confounding factor, which opens up the potential for continued use of Affectiva in research related to communication.

The use of technology as a clinical tool also has the potential to increase efficiency of clinicians [44] by reducing time taken for assessment analysis. Affectiva on iMotions is easy to set up, requires minimal technical skill, and provides immediate feedback on the use of facial expression [23]. This reduces time required for clinicians to conduct the assessment and analyze the results, as real-time data is provided by Affectiva. Lastly, Affectiva is a non-invasive tool that people with TBI could use independently as it provides direct and understandable feedback to people with TBI. Affectiva provides standardized quantifying data in the form of numeric scores for facial expression [23]. This could be used to measure change during the therapy and recovery period, and track progress. It could also function as a sensitive measure to determine treatment effectiveness.

Furthermore, future studies could evaluate different facial expressions during communication, and integrate different data using the iMotions software to gain a clearer picture of the cognitive impairments people have after TBI. The iMotions software has the capability of combining facial coding with other biosensors such as eye tracking [25], to quantify people’s attentional processes and behaviors, or electrodermal activity, to quantify emotional arousal [45]. Given there are strong correlations between attentional and emotional regulation impairments after TBI and social communication difficulties [46,47], integration of facial analysis, eye tracking and electrodermal activity could provide a more comprehensive objective assessment to guide and manage future therapy interventions.

## 5. Conclusions

This was the first study conducted to investigate the use of facial expression during a communication task by individuals with TBI using Affectiva. Affectiva detected differences between participants with TBI and control participants, with higher engagement on both tasks and greater smiling on one task found for participants with TBI. A key strength of the study design was that the TBI and control participants were well-matched according to demographics. However, there were other factors such as the positioning of the participants and the wearing of accessories that impacted on the automated facial expression analysis, and therefore influenced our findings. A key contribution of this study has been developing recommendations about future data collection to ensure high quality samples for analysis using Affectiva or similar technology. Future research could replicate this study with a standardized recording protocol using a larger and more diverse sample to investigate whether Affectiva can provide an objective and efficient clinical tool for assessment and intervention.

## Figures and Tables

**Table 1:** Demographics of participants with TBI.

Participant	Gender	Age	Years of Education	Language	Primary Language	Cause of Injury	PTA Days
TBI1	Male	49	17	Monolingual	English	MVA	41
TBI2	Male	48	10	Monolingual	English	Fall (height > 1 m)	14
TBI3	Male	32	16	Monolingual	English	Assault and/or fall (ground)	34
TBI4	Male	44	13	Monolingual	English	Fall (stairs)	19
TBI5	Male	40	17	Monolingual	English	MVA	55
TBI6	Male	28	15	Monolingual	English	MVA	64
TBI7	Male	26	18	Multilingual	Urdu	MVA	44
TBI8	Male	24	16	Bilingual	English	MVA	25
TBI9	Female	33	10	Bilingual	Singhalese	MVA	103
TBI10	Male	54	13	Monolingual	English	MVA	18

PTA = Post Traumatic Amnesia; TBI = Traumatic Brain Injury; MVA = Motor Vehicle Accident.

**Table 2:** Demographics of controls.

Participant	Gender	Age	Years of Education	Language	Primary Language
C1	Male	44.3	16	Multilingual	English
C2	Male	42.2	18	Monolingual	English
C3	Male	47.5	12	Monolingual	English
C4	Male	57.5	14	Monolingual	English
C5	Male	31.7	18	Monolingual	English
C6	Male	23	16	Monolingual	English
C7	Male	23.3	15	Monolingual	English
C8	Female	33.7	12	Monolingual	English
C9	Male	31.2	16	Multilingual	English
C10	Male	48.1	16	Monolingual	English

**Table 3:** Example of qualitative notes using an observation guide.

TBI/Control Participant	Rating 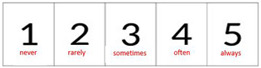			Describing the Context	Impression	Is the Use of Facial Expression Socially Appropriate?
	Engagement (Rating 1–5)	Smile (Rating 1–5)	Brow Furrow (Rating 1–5)			
TBI10	2	1	1	Brain injury story—talked about his accident → reported the accident to the police, waiting for police report. Situation: disheartening, unable to do much at the moment.	Startled facial expression (raised eyebrows), maintained throughout the video/expression did not change. Did not blink at all. However, used eye contact, body posture (leaning forward), and gesture to show engagement. Answered the interviewer’s question appropriately.	Inappropriate. The participant maintained a fixed facial expression throughout the 1 min conversation.

**Table 4:** Results generated by Affectiva of TBI participants and control participants on personal event narrative.

**TBI Participants**	**Engagement Time Percentage**	**Smile Time Percentage**	**Brow Furrow Time Percentage**
TBI1	48.78	0	12.7
TBI2	65.88	0	1.95
TBI3	85.75	47.6	2.95
TBI4	0	0	0
TBI5	46.27	0	3.63
TBI6	60.04	87.89	5.17
TBI7	17.55	19.22	0
TBI8	69.51	75.06	0
TBI9	46.23	58.91	7.05
TBI10	4.03	0	0
**Control Participants**	**Engagement Time Percentage**	**Smile Time Percentage**	**Brow Furrow Time Percentage**
C1	2.07	1.12	0.03
C2	51.93	0	69.44
C3	32.07	1.06	0.09
C4	1.32	0.51	0.07
C5	1.50	0	0.86
C6	1.78	0	0.12
C7	4.39	0.83	0.17
C8	0	0	0
C9	3.72	3.58	0
C10	1.84	2.75	4.31

**Table 5:** Results generated by Affectiva of TBI participants and control participants on story narrative retell.

**TBI Participants**	**Engagement Time Percentage**	**Smile Time Percentage**	**Brow Furrow Time Percentage**
TBI1	57.81	1.69	8.81
TBI2	72.28	7.87	26.41
TBI3	92.31	83.20	1.39
TBI4	1.83	0.83	0.88
TBI5	73.03	1.65	1.65
TBI6	51.65	47.00	8.43
TBI7	31.10	28.58	3.73
TBI8	51.54	49.89	0
TBI9	30.54	40.89	4.05
TBI10	83.53	0.41	0.19
**Control Participants**	**Engagement Time Percentage**	**Smile Time Percentage**	**Brow Furrow Time Percentage**
C1	1.93	0.08	0
C2	10.41	2.93	13.44
C3	25.23	0.15	0.56
C4	21.92	13.64	1.02
C5	0.58	0.16	0.28
C6	7.01	0.26	0.38
C7	0.39	0	0.01
C8	29.59	4.55	0.50
C9	0.38	0.38	0
C10	4.95	12.46	0.66

**Table 6:** Comparison of TBI and control participants on personal event narrative.

Facial Measures	TBI Participants (n = 10)	Control Participants (n = 10)	Statistical Significance
Engagement time percentage	Median: 47.53 Range: 0.00–85.75	Median: 1.96 Range: 0.00–51.93	*p* = 0.011 * U = 82.50
Smile time percentage	Median: 9.61 Range: 0.00–87.89	Median: 0.67 Range: 0.00–3.58	*p* = 0.481 *U* = 60.00
Brow furrow time percentage	Median: 2.45 Range: 0.00–12.70	Median: 0.11 Range: 0.00–69.44	*p* = 0.739 *U* = 55.00

* Statistically significant result (*p* < 0.05).

**Table 7:** Comparison of TBI and control participants on story narrative retell.

Facial Measures	TBI Participants (n = 10)	Control Participants (n = 10)	Statistical Significance
Engagement time percentage	Median: 54.73 Range: 1.83–92.31	Median: 5.98 Range: 0.38–29.59	*p* ≤ 0.001 * U = 93.00
Smile time percentage	Median: 18.23 Range: 0.41–83.20	Median: 0.32 Range: 0.00–13.64	*p* = 0.015 * U = 82.00
Brow furrow time percentage	Median: 2.69 Range: 0.00–26.41	Median: 0.44 Range: 0.00–13.44	*p* = 0.052 *U* = 76.00

* Statistically significant result (*p* < 0.05).

**Table 8:** Comparison between narrative tasks for the TBI group.

Facial Measures	Personal Event Story	Cinderella Story	Statistical Significance
Engagement time percentage	Median: 47.53 Range: 0.00–85.75	Median: 54.73 Range: 1.83–92.31	*p* = 0.386 *Z* = 0.866
Smile time percentage	Median: 9.61 Range: 0.00–87.89	Median: 18.23 Range: 0.41–83.20	*p* = 0.799 *Z* = 0.255
Brow furrow time percentage	Median: 2.45 Range: 0.00–12.70	Median: 2.69 Range: 0.00–26.41	*p* = 0.767 *Z* = 0.296

**Table 9:** Comparison between narrative tasks for the control group.

Facial Measures	Personal Event Story	Cinderella Story	Statistical Significance
Engagement time percentage	Median: 1.96 Range: 0.00–51.93	Median: 5.98 Range: 0.38–29.59	*p* = 0.878 *Z* = −0.153
Smile time percentage	Median: 0.67 Range: 0.00–3.58	Median: 0.32 Range: 0.00–13.64	*p* = 0.386 *Z* = 0.866
Brow furrow time percentage	Median: 0.11 Range: 0.00–69.44	Median: 0.44 Range: 0.00–13.44	*p* = 0.678 *Z* = −0.415

## Data Availability

Data is available on request from the author. The data are not publicly available for privacy reasons, as the dataset includes videos of participants.

## Appendix A. Qualitative Observation Form

**Table:**

TBI Participant/Control	Rating			Describing the Context	Impression	Is the Use of Facial Expression Socially Appropriate?
	Engagement (Rating 1–5)	Smile (Rating 1–5)	Brow Furrow (Rating 1–5)			
**TBI Participant Brain Injury Story**						
TBI1	1	1	1	Brain injury story—cannot remember the incident, but remember events 3 weeks afterwards, remember the treatment, but cannot remember details	- Probably because of context of story retell? Not a happy event, therefore less expressive. - Important life event video, participant showed more positive emotions and was more expressive - Some eye contact, other times participant looked away while thinking	Appropriate.
TBI2	1	1	1	Brain injury story—talked about how he remembered being aggressive after coming out of coma, snapping at things, how he coped with it, and how it improved over time	- Monotone - Appropriate eye contact - Some gestures used	Appropriate facial expression in the context of story recount. Facial expression matched tone of voice, flat.
TBI3	3	3	1	Brain injury story—talked about what he remembered and his body functions	- Unsure where the interviewer was positioned but seemed like there is eye contact with interviewer - Used gestures - Description of his memory: “always like I’m there, I’m a third person looking down”.	Appropriate facial expression.
TBI4	2	1	1	Brain injury story—talked about how his injury happened - Situation: slipped downstairs because neighbour left the window open, stairs was slippery due to rain	- Not facing camera, might be looking at interviewer instead - Answered interviewer question appropriately - Used gestures	Appropriate facial expression according to context.
TBI5	2	1	1	Brain injury story—talked about what people told him - Situation: very general and brief, neutral/no strong emotions involved	- Required further prompting from interviewer - Very brief description about his brain injury story - Used gestures - Appropriate eye contact	Appropriate in terms on facial expression, but inappropriate in terms of conversation (impoverished)
TBI6	1	2	1	Brain injury story—unsure topic content - Situation: no context, did not know what to say, was confused	- A lot of pauses - Seemed confused, not much content/did not know what to say - Required further prompting from interviewer - Asked for help to answer question—“what do you think?”	Appropriate facial expression. Inappropriate. The participant’s conversation lacked content, was confused.
TBI7	2	2	1	Brain injury story—talked about what he remembered → cannot remember about the accident day, cannot remember that he went to the doctor or CT scan, but remembered events at the rehabilitation centre - Situation: recalling of events	- Monotonous voice - Used hand gesture when explaining - Smiled to indicate he finished talking - Difficult to figure out eye contact when you do not know where the person is sitting	Appropriate in this context—conversation topic did not involve strong emotions Monotonous voice, flat voice throughout
TBI8	4	3	1	Brain injury story—talked about his hospital experience/memories → peed in bed, on crutches, having lunch, talking to nurses and doctors - Situation: light-hearted	- Appropriate eye contact - Leaned forward and used gestures	Appropriate.
TBI9	4	3	1	Brain injury story—talked about her accident → could not eat, could not remember her husband, parents, only remembered her son - Situation: sad	- Answered the interviewer’s question (but required further prompting from interviewer) - Appropriate eye contact - Smiled when she talked about sad moments (but seemed appropriate)—downturned smile/smile when you’re talking about something sad - Dysarthria → might have caused her slurred speech	Appropriate in terms on facial expression, but inappropriate in terms of conversation (impoverished)
TBI10	2	1	1	Brain injury story—talked about his accident → reported the accident to the police, waiting for police report - Situation: disheartening, unable to do much at the moment	- Startled facial expression (raised eyebrows), maintained throughout the video/expression did not change - Did not blink at all - However, used eye contact, body posture (leaning forward), and gesture to show engagement - Answered the interviewer’s question appropriately	Inappropriate. The participant maintained a fixed facial expression throughout the 1 min conversation.
**Control Injury/Illness Story**						
C1	4	2	3	Injury story—talked about his hip replacement - Situation: retell, no strong emotions involved	- Answered the interviewer’s question appropriately - Tone seemed flat sometimes - Used hand gesture (on one occasion) - Has key moments when he used his facial expression to bring the interviewer in - Microexpressions, e.g., raising eyebrows	Appropriate.
C2	2	1	1	Injury story—described how he sprained his ankle - Situation: retell, no strong emotion involved	- Answered the interviewer’s question appropriately - Tone seemed flat - Used gestures (nodding, hand gestures)	Appropriate.
C3	3	2	1	Injury story—described how they found out about his meningitis and events at the hospital and how he had no TV for 4 days Situation: retell	- Story-telling tone, variation - Brow raising, head shaking to express himself - Facial dots detecting the cap - Side facing	Appropriate.
C4	1	2	1	Injury story—tackled by a jock, broken wrist Situation: retell, no strong emotion involved	- Used gestures - Slight smile at the end - Appeared to maintain eye contact with interviewer	Appropriate.
C5	3	2	3	Injury story—went to freshman camp, bitten by fleas/bed bugs all over, broke out into rashes/hives Situation: retell	- Emphasis of words “bitten ALL over” - Smiled at the end - Frowned when recalling, and when he talked about awful moments - Used gestures (head shaking)	Appropriate.
C6	2	1	1	Injury story—“cracked” his shoulders during a football drill (dislocation)	- Used gestures - Answered the interviewer’s question appropriately - At times, tone seemed flat, no strong emotions involved - Leaned back, laidback	Appropriate.
C7	3	4	2	Injury story—talked about his meningitis	- Minimal eye contact with interviewer, looked away when recalling events - Frowned when recalling event - Flat tone but varied facial expression	Appropriate.
C8	1	1	1	Injury story—talked about how she got diagnosed with type 1 diabetes	- Slight smile at the end (possibly to indicate that she finished talking) - Some eye contact with interviewer (not directly facing camera), looked away most of the time to think and talk	Appropriate.
C9	3	3	1	Injury story—talked about his accident, hospitalised and how he got stitched up without anaesthesia Situation: retell, no strong emotions involved	- Answered the interviewer’s question appropriately - Monotonous - Laughed at appropriate moments	Appropriate.
C10	2	1	1	Injury story—talked about hurting his back, went for physical therapy	- Answered the interviewer’s question appropriately - Unsure where interviewer is but participant appeared to maintain eye contact with interviewer - Used gestures	Appropriate.
**TBI Participant Cinderella Story**						
TBI1	2	2	1	Cinderella story	- Smiled at the end - Main content included, some details missing - Participant appeared to maintain eye contact with interviewer at times. Looked away when thinking.	Appropriate.
TBI2	1	1	1	Cinderella story	- Participant appeared to maintain eye contact with interviewer at times. Looked away when thinking. - Main content included - Described the animals as Cinderella’s pets - Monotonous voice	Appropriate.
TBI3	3	4	1	Cinderella story	- Laughed on a few occasions—when he made a mistake/at his own comments - Main content and some details included - Included personal opinion on some occasions - Used gestures	Appropriate.
TBI4	1	1	1	Cinderella story	- Not facing camera, but participant seemed to maintain eye contact with interviewer - Used gestures - Missing and some inaccurate content and details - Facial dots got lost momentarily	Appropriate in terms on facial expression, but inappropriate in terms of narrative retell.
TBI5	2	2	1	Cinderella story	- Used gestures - Smiled/laughed at the end - Minimal eye contact when recalling narrative story	Appropriate.
TBI6	2	2	3	Cinderella story	- Long pauses when recalling events - Missing details - Monotonous voice - Reduced rate of speaking	Appropriate in terms on facial expression, but inappropriate in terms of narrative retell.
TBI7	1	1	1	Cinderella story	- Missing details - Difficulty using specific words “something”, fairy godmother→ ‘magician” - Monotonous voice	Appropriate in terms on facial expression, but inappropriate in terms of narrative retell.
TBI8	3	1	1	Cinderella story	- Minimal eye contact when recalling narrative story - Main content and details included	Appropriate.
TBI9	2	2	1	Cinderella story	- Brief story retell (e.g., went to another party, danced with the man, while she came out she missed shoes, then she went home) - Smiled at the end—indication that she finished - Flat tone	Appropriate in terms on facial expression, but inappropriate in terms of conversation (impoverished).
TBI10	1	2	1	Cinderella story	- Startled facial expression (raised eyebrows) - Body posture—leaned forward - Smiled at the end of the narrative retell (but not throughout the story) - Unable to balance to cognitive load? - Monotonous voice	Inappropriate. The participant maintained a fixed facial expression throughout the narrative retell (except when he turned away on one occasion, and on another occasion, smiled when he finished).
**Control Cinderella Story**						
C1	2	2	1	Cinderella story	- Microexpression (brow raise, eyes) - Main content and details included - Flat tone - Smiled at the end	Appropriate.
C2	1	2	2	Cinderella story	- Brief description of the story - Minimal eye contact with interviewer - Monotonous voice - More intense person, brow furrow	Appropriate.
C3	1	1	1	Cinderella story	- Main content and details included - Intonation, story-telling voice	Appropriate.
C4	1	2	1	Cinderella story	- Some inaccurate information—instead of stepmother, participant said father’s sister and cousins - Monotonous voice - Smiled at the end	Appropriate.
C5	1	1	1	Cinderella story	- Main content and details included - Flat tone	Appropriate.
C6	1	2	1	Cinderella story	- Main content and details included - Leaned back and forward, half of the face not captured on camera - Monotonous voice	Appropriate.
C7	2	2	1	Cinderella story	- Main content and details included - Looked down most of the time - Smiled at the end	Appropriate.
C8	2	2	1	Cinderella story	- Main content and details included - Intonation during dialogues, e.g., “what are you crying for” - Minimal eye contact with interviewer - Smiled at the end	Appropriate.
C9	2	3	1	Cinderella story	- Main content and details included - Smiled at the end too - Generally smiley throughout	Appropriate.
C10	2	2	1	Cinderella story	- Main content included but details briefly described - Used gestures - Intonation - Maintained eye contact with interviewer	Appropriate.
